# Improvment of combined solar chimney power plant with gas power plant

**DOI:** 10.1038/s41598-023-38464-4

**Published:** 2023-07-11

**Authors:** Amin Mirzamohammad, Mohammad Eftekhari Yazdi, Arash Mirabdolah Lavasani

**Affiliations:** Department of Mechanical Engineering, Central Tehran Branch, Islamic Azad University, Tehran, Iran

**Keywords:** Mechanical engineering, Renewable energy

## Abstract

Recently, several researches have been done to improve the perfomance of solar chimney power plants (SCPP) and increase their low output power during hours when the solar radiation is limited. In this study, by combining a SCPP and a gas power plant, the output power is increased and the power output of the combined power plant can be gained at all hours of the day and night. Pipes are buried under the ground and the outlet hot gas from gas power plant flows through the buried pipes instead of being released into the atmosphere through the stacks. Flowing of hot gas through the buried pipes at the soil under the canopy increases the temperature of soil which is exposed to the solar radiation. Increasing of the soil temperature leads to the growth in the value of air temperature under the canopy. The air density reduces as the air temperature increases which leads to the increase of air velocity and output power. By applying the buried pipes, the output power is not zero during the hours when there is no radiation flux. The results for air temperature, heat loss and output power are studied in detail and it is shown that the use of buried pipes in which hot gas flows leads to the increase of the output power of SCPP by 554%, 208% and 125% at the radiation flux of 200 W/m^2^, 500 W/m^2^ and 800 W/m^2^, respectively.

## Introduction

The air pollution and the limited sources of fossil fuels are the major problems that humans have faced in recent decades. Many solutions have been proposed by researchers to reduce the concentration of pollutants^1–4^ and to replace fossil fuels sources with the other energy sources^5–8^. Solar power is identified as one of the new resources of energy and the power can be generated from that by employing various equipment such as solar chimney Power Plant (SCPP). It contains a tall chimney and a collector to capture the energy of solar^9,10^.

Many Authors investigated the generation of power in SCPP experimentally^11–13^ and numerically^14–18^. The efficiency of SCPP is strongly associated with the environmental conditions and geometric parameters. Extensive studies have been conducted to examine the impacts of environmental factors such as wind speed^19^, ambient air temperature^20^, soil porosity^21^, solar flux^22^ and site altitude^23^ on the enactment of SCPP. Along with the environmental conditions of the place where the power plant is built, the geometric parameters of the SCPP also strongly affect the efficiency and performance of the power plant. The effects of geometric parameters i.e. collector slope, chimney diverging angle, collector height and radius, absorber plate diameter, chimney radius and height on power plant efficiency were investigated in detail^24–27^. Keshari et al.^28^ numerically studied the effects of collector angle on the operation of SCPP. The collector inclination angle was assumed to be changed from 0° to 30°. They found that the maximum value of output power was achieved at collector angel equal to 6°.

One of the main challanges of using solar energy is that it is not available at all hours of the day. In order to be able to use solar energy sustainably, the problem of its permanent unavailability must be solved. One way that has received considerable attention is to apply an energy storage system with SCPP^29–32^. In this combined system, the solar radiation in the available hours, in addition to generating power in SCPP, is also stored in the energy storage system so that the stored energy can be employed to generate power during the times that the solar radiation is not available. The other solution to product sustainable power is to use a SCPP that required heat can be supplied from another source in addition to the solar radiation. In some gas power plant, the hot outlet gas from turbine is released into the atmosphere which leads to the air pollution and wasting energy^33,34^. Researchers^35–38^ tried to use the outlet hot gas from gas power plants in a SCPP to increase output power as well as product sustainable power. They employed different techniques to use the energy of the exhaust gas from the gas power plant in SCPP. In some studies, ducts have been installed under the collector for the flow of hot gas to increase the air temperature under the collector. In some other work, hot gas is discharged under the collector to merge with the air which leads to the increase of the air temperature. Using both techniques can cause problems. In the first technique, a heterogeneous temperature distribution is created inside SCPP in which the air temperature is higher near the ducts and the air temperature decreases by moving away from the ducts. In the second technique, the air under the collector is very polluted due to the direct injection of the exhaust gas from the gas turbine, and therefore the activity in this area will be associated with problems.

In this study, the outlet hot gas from gas power plant flows through the buried pipes. There are many advantages to this type of configuration. The contaminant does not enter the area under the canopy and activities such as agriculture can be continued under it^39^. Also, by burying the pipes at the sufficient depth of the ground, uniform heat transfer happens on the ground surface. In the configurations previously examined, the contaminant enters the area below the collector and also the air temperature around the duct below the collector is higher and a uniform temperature distribution cannot be found. The limitations in previous studied configurations are removed by employing buried pipes. In this study, the output power of SCPP is obtained in two cases where there are pipes underground and there are not pipes underground.

## Definition of problem

**Figure 1:**
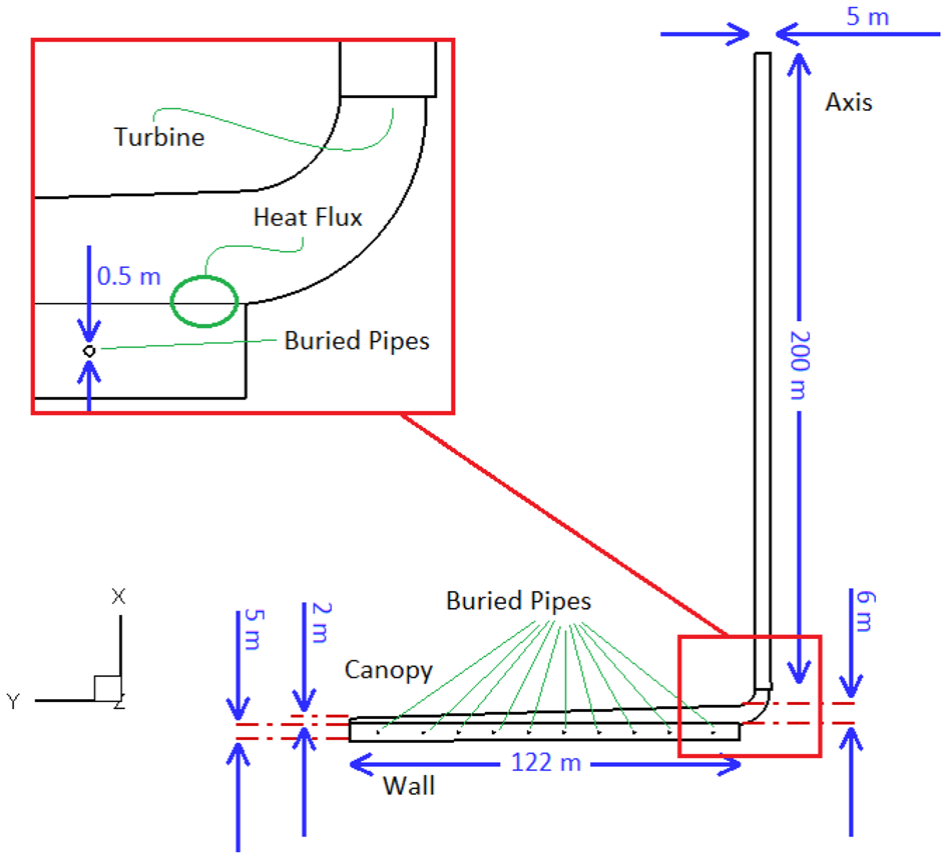
The geometry of SCPP and the boundary conditions.

There are several lessens regarding transition away from fossil fuels toward renewables^40–42^. A SCPP similar to Spanish prototype^10^ is evaluated in this study. The SCPP includes of a chimney which is connected smoothly to a collector. The soil and stones under the collector is considered as energy storage layer where the buried pipes are located. Figure 1 dispalys the configuration of SCPP with the proposed buried pipes. Thermal conductivity, specific heat capacity and density of soil are considered to be λ = 0.78 W/m K, C_p_ = 2016 J/kg K and ρ = 1700 kg/m^3^, respectively^21^. The soil is considered to be like porous media and the fluid flow behavior in that is obtained by employing Brinkman–Forchheimer-extended Darcy model^43^. The depth of the energy storage layer is equal to 5 m and the pipes are buried at a depth of 2.5 m. The lower side of the energy layer is considered to be the wall with a constant temperature equal to 300 K. Canopy is a sloping wall that it is located at the height of 2 m at the inlet and at the height of 6 m in the center of SCPP. The thin layer of soil (0.001 m) at the top of the energy storage layer is assumed as heat source with different values of heat flux to simulate solar radiation^44^. The turbine is considered as a fan and it is modeled similar to the pressure drop boundary condition^45^.

**Figure 2:**
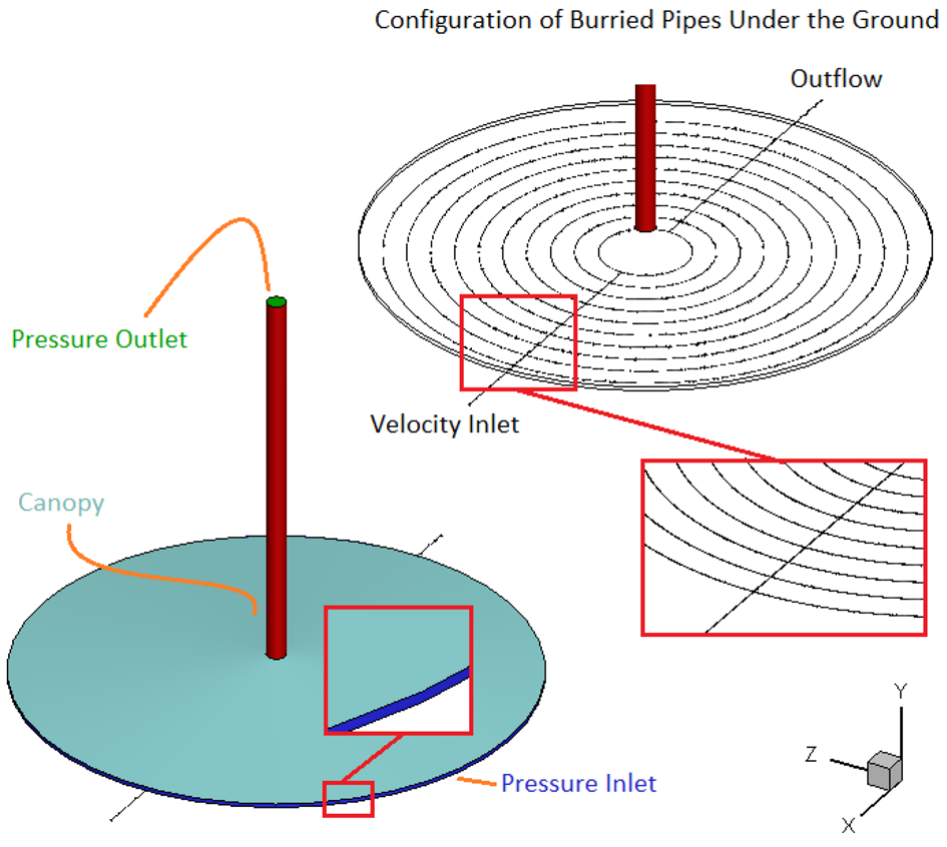
The configuration of buried pipes as well as boundary conditions.

Figure 2 shows that the inlet and outlet of SCPP are assumed atmospheric pressure. Unlike the wall of chimney, which is assumed to be adiabatic, heat can be transferred through the canopy. The convection boundary condition is assumed for canopy with the convection coefficient and surrounding air temperature of 10 W/m^2^ K and 293 K, respectively. Figure 2 shows the configuration of buried pipes. It can be found that the hot gas enters the circular buried pipes through a pipe with an inlet velocity boundary condition. After completing the semicircular path, the gas exists through a pipe with outflow boundary condition at the outlet of that. The mass flow rate of outlet hot gas from a gas power plant is considered to be 640.65 kg/s. It means that the flow rate of hot gas is 522.98 m^3^/s. Since the diameter of the buried pipes is equal to 0.5 m and their number is equal to 10 rows, so the gas velocity in the buried pipe is 266.49 m/s. The temperature of hot gas at the inlet of pipe is 816.9 K. Because the velocity of the hot gas in the pipes is very high, it can be assumed that the gas temperature does not decrease by passing through the buried pipes, and therefore the constant temperature boundary condition can be assumed on the wall of the buried pipes and the 2D numerical simulation can be performed.

The assumption of a constant temperature boundary condition on the wall of the buried pipes is a simplification that can potentially limit the accuracy of a 2D numerical simulation of the system. In reality, the temperature of the surrounding soil can vary with depth and time, and the thermal conductivity and diffusivity of the soil can also vary with moisture content, porosity, and other factors.

In addition, the 2D numerical simulation assumes a homogeneous soil profile and does not account for the effects of soil heterogeneity or groundwater flow. These factors can also affect the accuracy of the simulation and introduce uncertainties in the predicted temperature distribution and heat transfer rates.

A 3D numerical simulation can provide more accurate results by accounting for the effects of soil heterogeneity and groundwater flow, as well as the three-dimensional nature of the heat transfer processes. However, a 3D simulation also requires more computational resources and may be more complex to set up and calibrate.

Overall, the choice between a 2D and 3D numerical simulation depends on the specific characteristics of the system being studied and the desired level of accuracy. While a 3D simulation may provide more accurate results, a 2D simulation can still be a useful tool for predicting the overall performance of the system and identifying key design parameters. It is important to carefully evaluate the assumptions and limitations of the numerical model and validate the results with experimental data to ensure the accuracy and reliability of the simulation.

## Mathematical formulation

Numerical technique of CFD was extensively used for the modeling of engineering problems^46–55^ Computational domain is divided into two areas and different governing equations should be solved in each of which. Navier–Stokes equation should be solved to examine the air flow in the chimney and the area below canopy^56–60^. On the other hand, the Brinkman–Forchheimer-extended Darcy model should be employed to investigate the fluid flow and heat transfer in the energy storage layer. The Brinkman-Forchheimer-extended Darcy model is a commonly used mathematical model for describing fluid flow in porous media, including soil. This model includes additional terms that account for inertial and viscous effects that are not accounted for in the traditional Darcy model.

The accuracy of the Brinkman-Forchheimer-extended Darcy model in describing fluid flow behavior in porous media depends on a number of factors, including the specific characteristics of the porous media (e.g., pore size distribution, porosity), the fluid properties (e.g., viscosity, density), and the flow conditions (e.g., velocity, pressure gradient).

In general, the Brinkman-Forchheimer-extended Darcy model can provide more accurate predictions of fluid flow behavior in porous media than the traditional Darcy model, particularly in cases where the flow velocity is relatively high or where the pore size distribution is highly heterogeneous. However, the accuracy of the model is still dependent on the assumptions and simplifications used in its formulation, and validation with experimental data is necessary to determine its accuracy in a specific case.

Overall, the use of the Brinkman-Forchheimer-extended Darcy model can be a valuable tool for improving the accuracy of fluid flow predictions in porous media, including soil. However, careful consideration of the specific conditions and limitations of the model is necessary to ensure accurate results.

In these equations, the subscripts 1 indicate the parameters in the area out of soil. The air density is considered as a function of temperature which is modeled by applying Boussinesq’s approximation.

1 $$\frac{{\partial \rho u_{1} }}{\partial x} + \frac{{\partial \rho v_{1} }}{\partial y} = 0$$

2 $$\frac{{\partial \left( {\rho u_{1} u_{1} } \right)}}{\partial x} + \frac{{\partial \left( {\rho v_{1} u_{1} } \right)}}{\partial y} = - \frac{{\partial P_{1} }}{\partial x} + \mu \left( {\frac{{\partial^{2} u_{1} }}{{\partial x^{2} }} + \frac{{\partial^{2} u_{1} }}{{\partial y^{2} }}} \right)$$

3 $$\frac{{\partial \left( {\rho u_{1} v_{1} } \right)}}{\partial x} + \frac{{\partial \left( {\rho v_{1} v_{1} } \right)}}{\partial y} = - \frac{{\partial P_{1} }}{\partial y} + \rho g\beta \left( {T - T_{\infty } } \right) + \mu \left( {\frac{{\partial^{2} v_{1} }}{{\partial x^{2} }} + \frac{{\partial^{2} v_{1} }}{{\partial y^{2} }}} \right)$$

4 $$\frac{{\partial \left( {\rho Cu_{1} T} \right)}}{\partial x} + \frac{{\partial \left( {\rho Cv_{1} T} \right)}}{\partial y} = \lambda_{1} \left( {\frac{{\partial^{2} T}}{{\partial x^{2} }} + \frac{{\partial^{2} T}}{{\partial y^{2} }}} \right)$$

5 $$\frac{{\partial \left( {\rho ku_{1i} } \right)}}{{\partial x_{i} }} = \frac{\partial }{{\partial x_{j} }}\left( {\left( {\mu + \frac{{\mu_{t} }}{{\sigma_{k} }}} \right)\frac{\partial k}{{\partial x_{i} }}} \right) + G_{k} + G_{b} - \rho \varepsilon$$

6 $$\frac{{\partial \left( {\rho \varepsilon u_{1i} } \right)}}{{\partial x_{i} }} = \frac{\partial }{{\partial x_{j} }}\left( {\left( {\mu + \frac{{\mu_{t} }}{{\sigma_{\varepsilon } }}} \right)\frac{\partial \varepsilon }{{\partial x_{j} }}} \right) + C_{1\varepsilon } \left( {G_{k} + C_{3\varepsilon } G_{b} } \right) - C_{2\varepsilon } \rho \frac{{\varepsilon^{2} }}{k}$$

where u, v, ρ, P and μ are velocity in x direction, velocity in y direction, density of air, pressure and viscosity, respectively. g, β, T, C and λ_1_ are gravity, thermal expansion coefficient, temperature, heat capacity and thermal conductivity of air, respectively^33^.

The energy storage is considered porous media and Brinkman–Forchheimer-extended Darcy model is employed^34^ to investigate the fluid flow and heat transfer in it.

7 $$\frac{{\partial \rho u_{2} }}{\partial x} + \frac{{\partial \rho v_{2} }}{\partial y} = 0$$

8 $$\frac{1}{{\varphi^{2} }}\left( {\frac{{\partial \left( {\rho u_{2} u_{2} } \right)}}{\partial x} + \frac{{\partial \left( {\rho v_{2} u_{2} } \right)}}{\partial y}} \right) = - \frac{{\partial P_{2} }}{\partial x} + \frac{\mu }{\varphi }\left( {\frac{{\partial^{2} u_{2} }}{{\partial x^{2} }} + \frac{{\partial^{2} u_{2} }}{{\partial y^{2} }}} \right) - \left( {\frac{\mu }{K} + \frac{{\rho C_{F} }}{\sqrt K }\left| {u_{2} } \right|} \right)u_{2}$$

9 $$\frac{1}{{\varphi^{2} }}\left( {\frac{{\partial \left( {\rho u_{2} v_{2} } \right)}}{\partial x} + \frac{{\partial \left( {\rho v_{2} v_{2} } \right)}}{\partial y}} \right) = - \frac{{\partial P_{2} }}{\partial y} + \rho g\beta \left( {T - T_{\infty } } \right) + \frac{\mu }{\varphi }\left( {\frac{{\partial^{2} v_{2} }}{{\partial x^{2} }} + \frac{{\partial^{2} v_{2} }}{{\partial y^{2} }}} \right) - \left( {\frac{\mu }{K} + \frac{{\rho C_{F} }}{\sqrt K }\left| {v_{2} } \right|} \right)v_{2}$$

10 $$\frac{{\partial \left( {\rho Cu_{2} T} \right)}}{\partial x} + \frac{{\partial \left( {\rho Cv_{2} T} \right)}}{\partial y} = \lambda_{2} \left( {\frac{{\partial^{2} T}}{{\partial x^{2} }} + \frac{{\partial^{2} T}}{{\partial y^{2} }}} \right)$$

where φ is the porosity of soil and λ_2_ is the effective thermal conductivity of soil. K and C_F_ are permeability and inertial coefficient of soil. ANSYS software is used for the simulation of the air in the selected model.

The output power of SCPP is calculated from solving the Eq. 11. ∆P is fluid pressure drop as it passes through the turbine. V is volumetric air flow passing through the chimney. The efficiency of turbine (η_t_) is considered to be equal to 80%^42,43^.

11 $$W_{t} = \eta_{t} \cdot \Delta P \cdot V$$

The performance of the solar chimney power plant (SCPP) depends on a variety of factors, including the design and operating conditions of the system, as well as the solar radiation input. The heat flux values used to simulate solar radiation can significantly affect the performance of the SCPP, as they directly impact the amount of heat that is absorbed by the collector and the resulting temperature difference that drives the airflow through the chimney.

The accuracy of the heat flux values used to simulate solar radiation is therefore critical for predicting the performance of the SCPP. Inaccurate or uncertain values can lead to significant errors in the predicted temperature gradients and airflow rates, which can in turn affect the power output and efficiency of the system.

Various methods can be used to estimate the heat flux values for solar radiation, including measurements from on-site weather stations and satellite-based data. However, these methods can also introduce uncertainty and variability in the input data.

Overall, the sensitivity of the SCPP performance to the heat flux values used to simulate solar radiation highlights the need for accurate and reliable data sources, as well as careful consideration of the uncertainties and limitations of the input data in the design and operation of the system.

**Figure 3:**
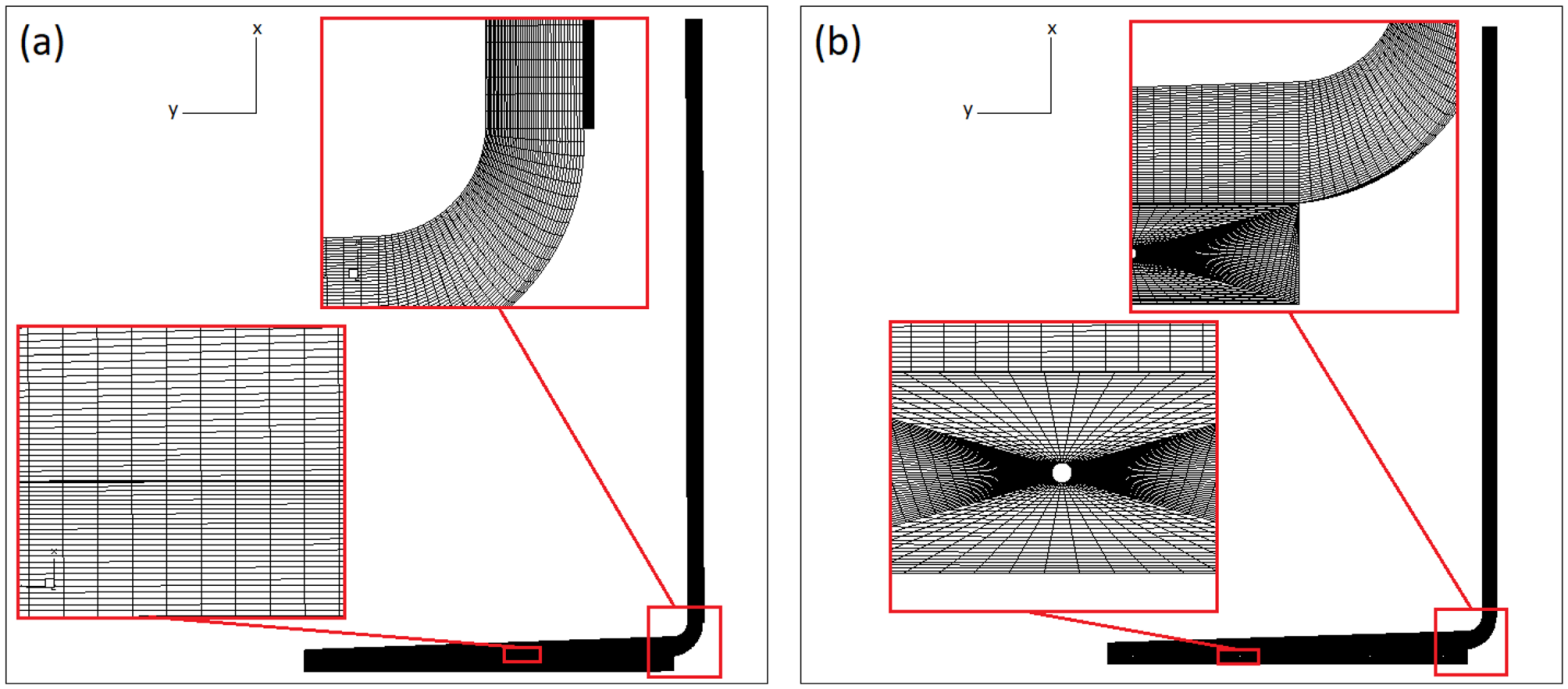
Grid distribution in the computational domain.

The second order upwind scheme and SIMPLE algorithm are chosen to discretize the governing equations and to derive a condition for pressure. Figure 3 shows the generation of grid in the computational domain for the case without buried pipes (a) and the case with buried pipes (b). The grid independency of the results is examined and it can be found that independent results are achieved for the case without the buried pipes and the case with the buried pipes where the computational nodes number are 586,974 and 732,412, respectively.

## Results

Accuracy of simulation must be checked before the investigation of obtained results. For this purpose, the boundary conditions of the problem are changed to compare the obtained results with the available data. The radiation flux is assumed to be 1000 W/m^2^ and the turbine is removed from SCPP so that the air flow is not experience a local pressure drop. These conditions are exactly the same as the conditions under which the results were obtained^10^. Table 1 compares the results obtained in this study for the air velocity in the chimney and also the increase of air temperature in the power plant with the available data^10^.

**Table 1:** Velocity and temperature of air in SCPP as well as those obtained by Haaf^10^.

Results	Temperature of air (C)	Velocity of air (m/s)
Measured data^10^	20	15
Obtained results	22.98	16.01

Comparison of results indicate that the errors of air temperature and air velocity are 15% and 7%, respectively. The results are obtained in two cases where the pipes are buried under the ground and they are not buried. Numerical errors arise from the discretization and approximation of the mathematical model in the numerical simulation. These errors can include errors in the discretization of the spatial and temporal domains, errors in the approximation of the governing equations, and errors in the numerical methods used to solve the equations. Numerical errors can be minimized by using smaller time and space steps, higher-order numerical methods, and more advanced numerical techniques.

**Figure 4:**
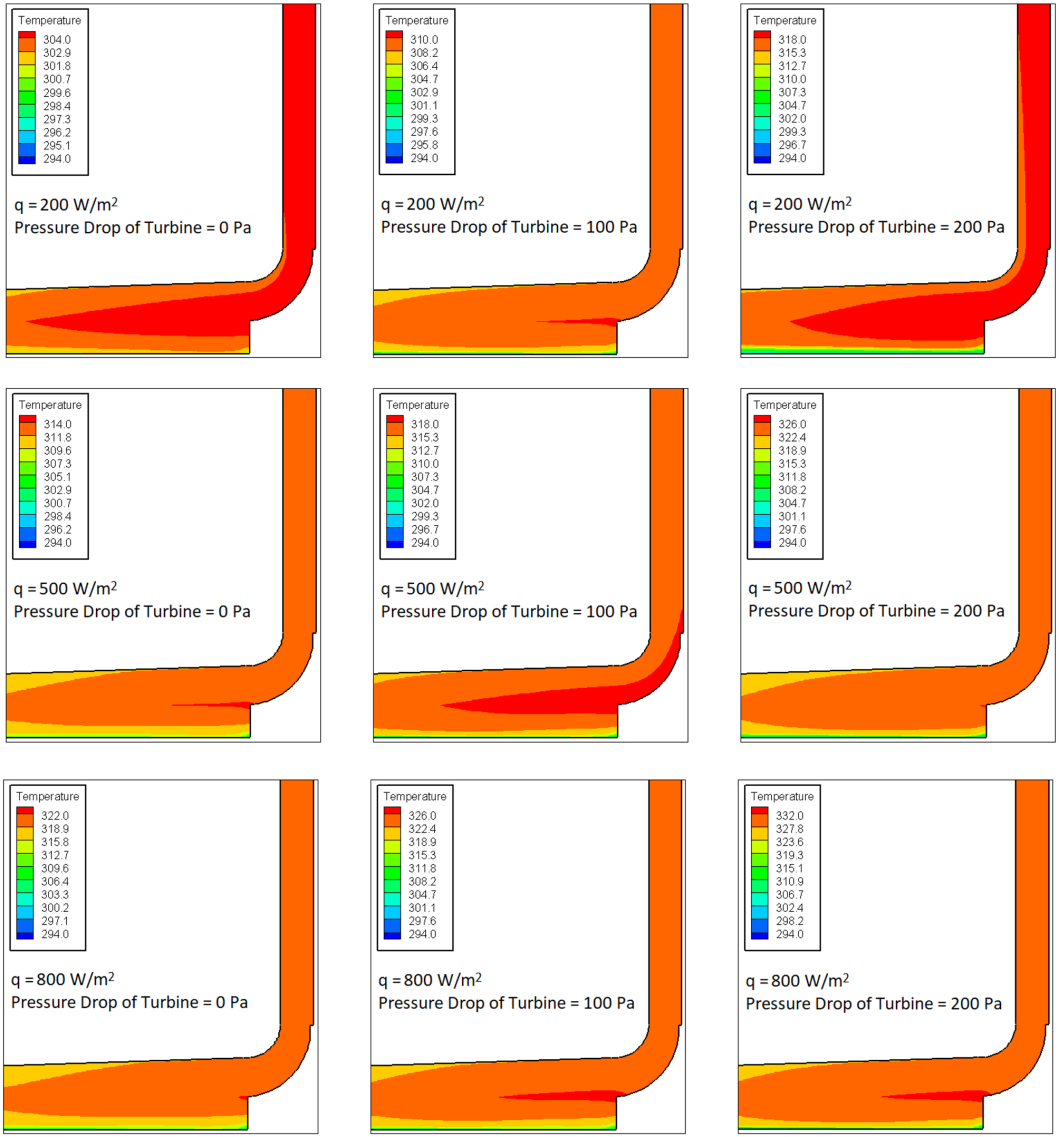
Contours of temperature in SCPP without buried pipes.

**Figure 5:**
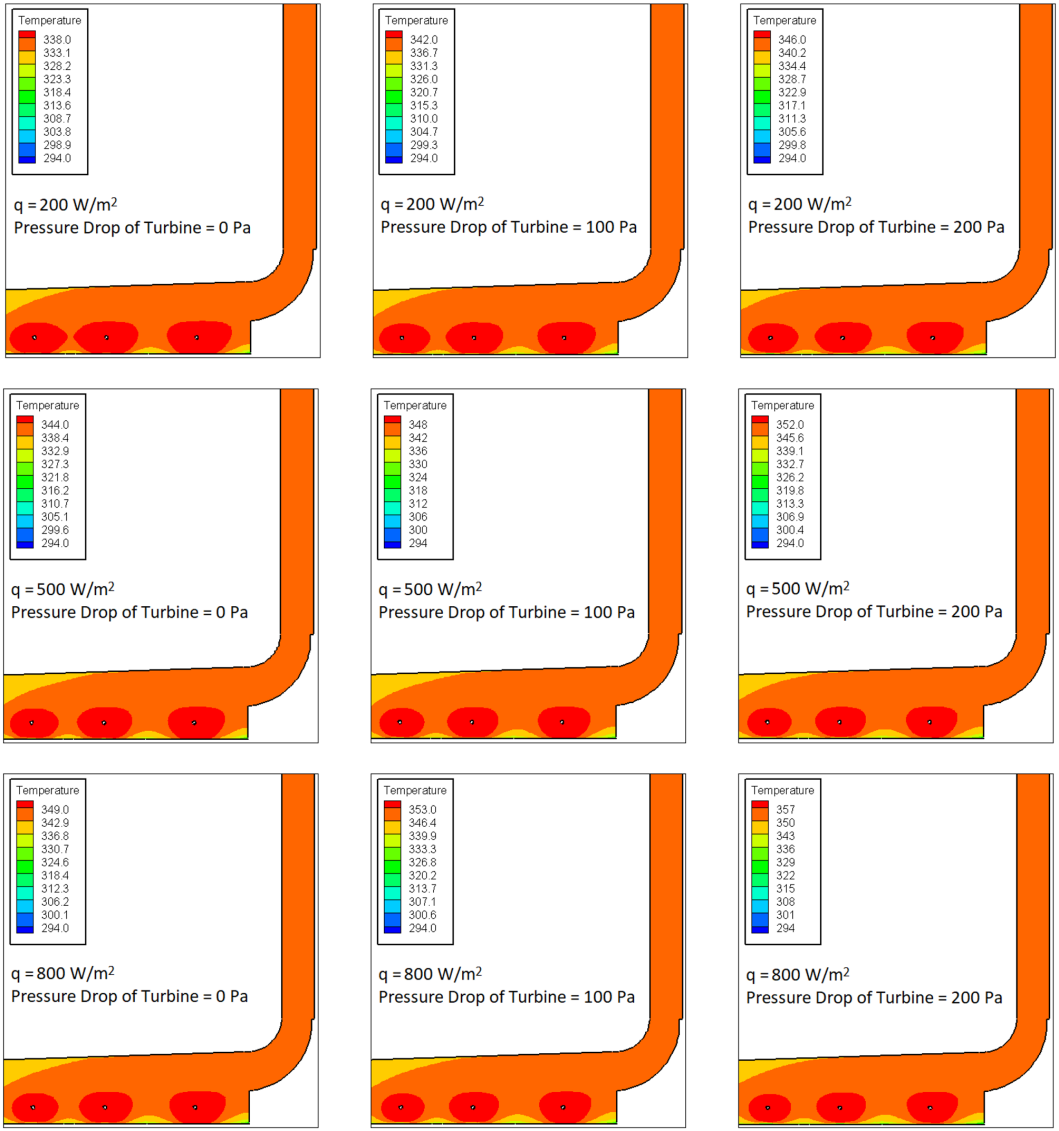
Contours of temperature in SCPP with buried pipes.

The impacts of solar radiation as well as pressure change of turbine on the variation of temperature in the computational domain is investigated for the case without buried pipes (Fig. 4) and the case with buried pipes (Fig. 5). Figure 4 shows that as the radiation flux increases, the maximum temperature measured in the SCPP increases. This is predictable because the increase in the rate of heat transfer is found by rising of the radiation flux, which leads to the increase in the value of air temperature. Besides, as the pressure drop of the turbine increases, the air temperature in SCPP rises. As the pressure drop of the turbine rises, the obstacle is created in the path of fluid flow. Therefore, as the pressure drop increases, the speed of the air moving along the chimney decreases, which causes the air to be in contact with the collector for a longer period of time, and thus the air temperature increases.

Figure 5 illustrates the air temperature in SCPP with the buried pipes. The maximum temperature equal to 816.9 K can be found on the walls of the buried pipes. In Fig. 5, the upper and lower temperature ranges for temperature contours are determined based on the lowest temperature of the air below the canopy and the highest temperature of the air below the canopy. The high temperature zone in the energy storage layer is formed centrally around the buried pipes in such a way that with a small distance from the buried pipes, the measured temperature decreases sharply. In all values of pressure drop and solar radiation of turbine, it is observed that the air temperature in SCPP with buried pipes is higher than that in SCPP without buried pipes. In the case study with buried pipes, it can also be found that increasing the radiation flux and pressure drop of turbine increases the maximum measured air temperature in SCPP.

**Figure 6:**
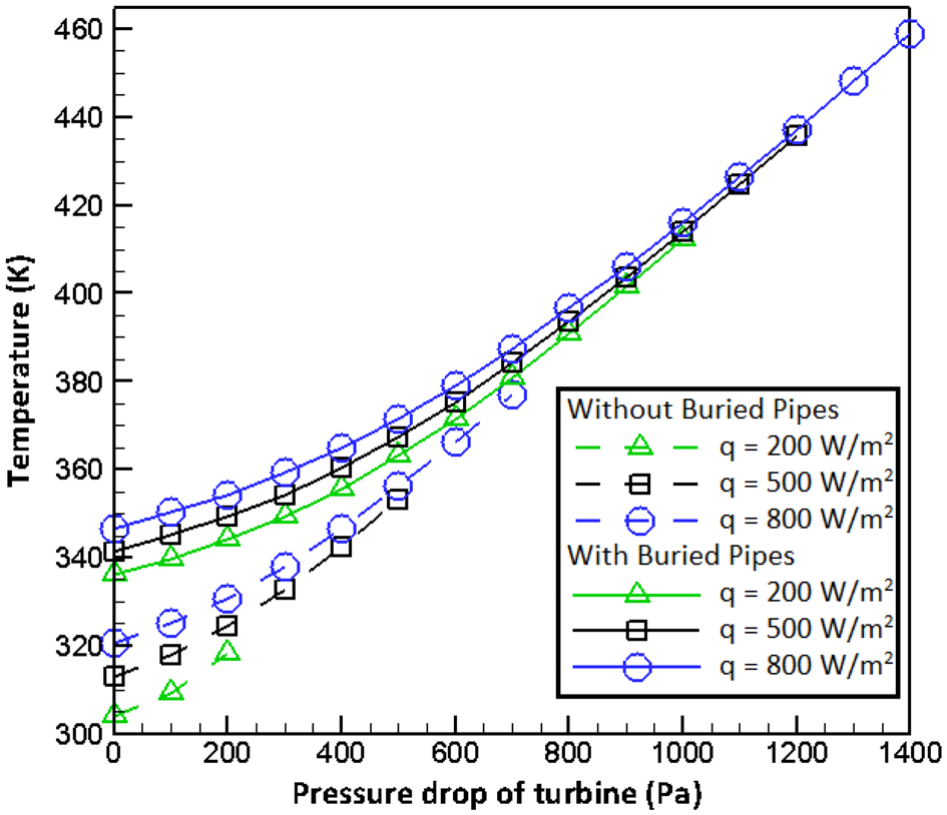
Effects of radiations as well as turbine pressure drops on the outlet temperature from SCPP.

In order to make a more complete quantitative comparison, the temperature is studied at a specific area of the computational domain instead of the temperature contours. As demonestrated in Fig. 6, the average air temperature at the outlet of chimney is selected for comparison. Figure 6 shows that burying pipes in which hot gas flows causes to increase the air temperature at the chimney’s outlet. It can be found that the fluid temperature at the power plant outlet growths by increasing in turbine pressure drop, and the changes of fluid temperature with regarding to the turbine pressure drop is higher where the pressure drop of turbine is higher. It can also be found that with increasing radiation flux, the air temperature growths at the outlet of SCPP. With increasing turbine pressure drop, the impacts of radiation flux on the outlet air temperature of the chimney becomes negligible.

**Figure 7:**
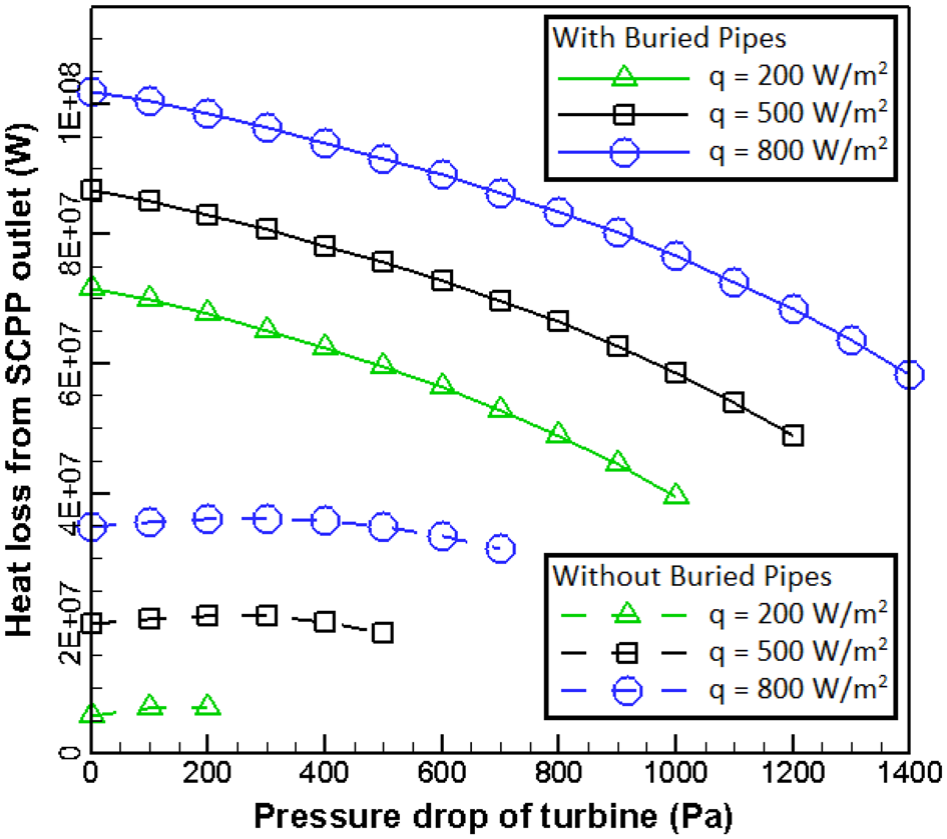
Heat loss at the exhaust of chimney for various pressure drops of turbine and solar radiation.

**Figure 8:**
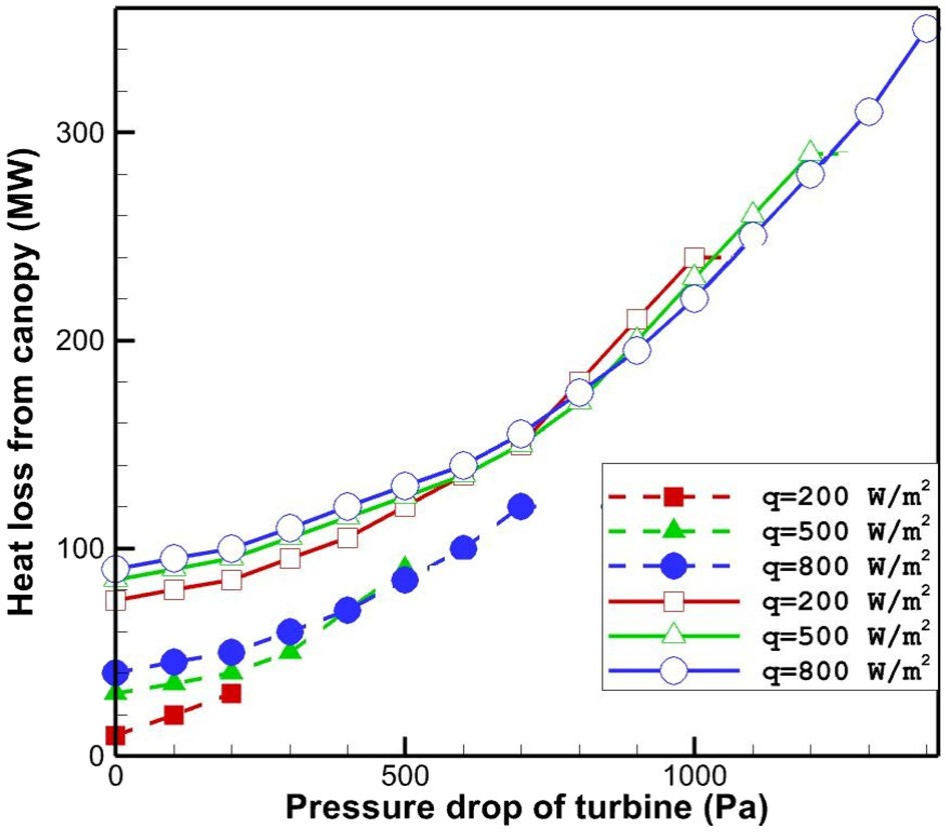
Lost heat through canopy for various pressure drops of solar radiation and turbine (dash line for unburied pipe and solid line for buried pipe).

One of the parameters considered in this study is heat loss from different parts of SCPP, which could highly influence on the performance of power plant. Heat losses from the outlet of power plant and canopy are shown in Figs. 7 and 8, respectively. As can be found in Fig. 7, studies on heat loss from the outlet of SCPP can be divided into two general parts. For the case without buried pipes, the heat loss growths as the radiation flux increases. But as the pressure ratio of turbine increases, the wasted heat from the outlet of chimney first rises and then decreases. For the case with buried pipes, the heat loss growths as the radiation flux increases and the pressure drop of turbine reduces.

Figure 8 shows the lost heat through the canopy for various values of pressure drop and solar radiation of turbine. It can be found that the lost heat through the canopy growth as the pressure drop of turbine increases. By ignoring the area related to the low pressure ratio, it can be concluded that by increasing the pressure drop of the turbine, the lost heat through the outlet of SCPP decreases but the lost heat through the canopy increases. The behavior of wasted heat through canopy relative to the variation of radiation flux is complex and it can be found that for low pressure drop values, the wasted heat increases with increasing in the value of radiation flux. In the high-pressure ratio values, the lost heat through canopy is first an rising function of the radiation flux and then with the growth in value of radiation it becomes a descending function of radiation flux.

**Figure 9:**
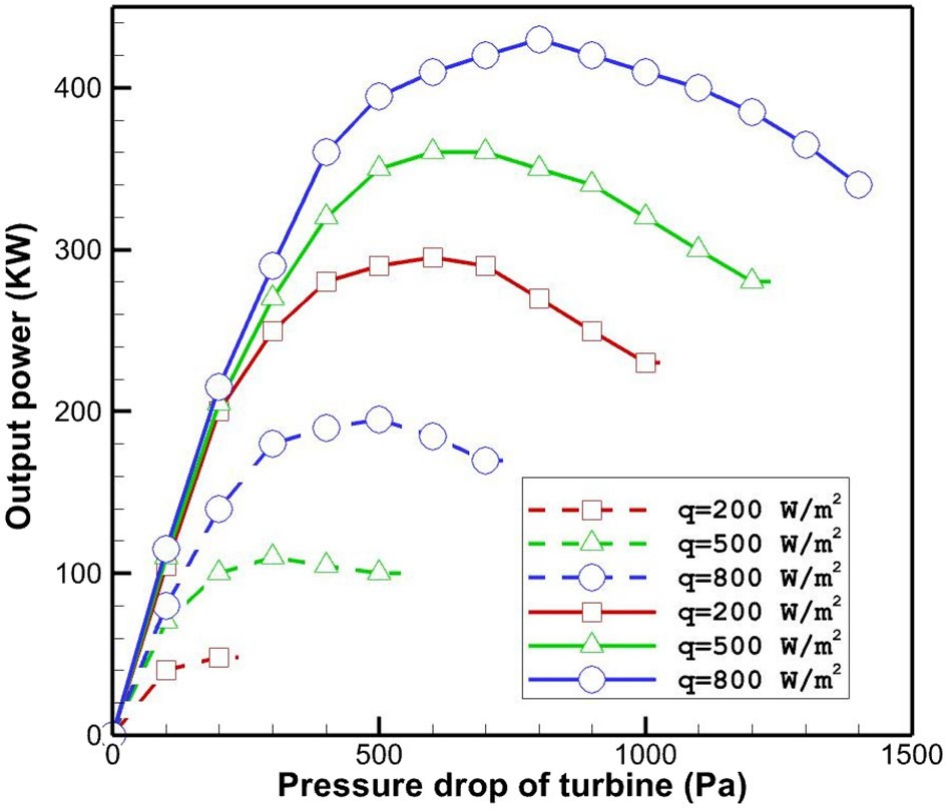
Output power from SCPP for the case without buried pipes (dash line) and the case with buried pipes (solid line).

Figure 9 shows that burying the pipes in which the outlet hot gas from the gas turbine flows increases the output power of SCPP. Burring the pipes under the ground leads to the increase in the output power of SCPP by 554%, 208% and 125% at the radiation flux of 200 W/m^2^, 500 W/m^2^ and 800 W/m^2^, respectively. Indeed, in the low radiation fluxes, the percentage of increase in power output of the power plant is higher by burying the pipes in which hot gas flows. Despite the large changes in the percentage of increase in output power for different values of solar radiation, it can be found that the growth in output power for radiation fluxes of 200 W/m^2^, 500 W/m^2^ and 800 W/m^2^ is equal to 251 kW, 246 kW and 242 kW, respectively. Thus, the rise in the output power of SCPP by using buried pipes containing hot gas flow is almost independent of the solar radiation.

The output power of a solar chimney power plant (SCPP) is driven by the temperature difference between the air inside the chimney and the ambient air outside, which creates a pressure difference that drives the airflow through the system. The pressure drop across the turbine is a key factor that affects the power output of the SCPP.

As the pressure drop across the turbine increases, the air velocity through the turbine also increases, which can lead to increased turbulence, frictional losses, and other effects that reduce the efficiency of the turbine. At some point, the pressure drop may become too high, and the turbine may stall or operate at a reduced efficiency, leading to a decrease in the power output of the SCPP.

In addition to the effects of turbine performance, there may be other factors that affect the power output of the SCPP at high pressure drops. For example, the pressure drop may also affect the flow behavior of the air in the chimney, such as the formation of vortices or other flow instabilities that can reduce the efficiency of the system.

Overall, the exact reasons for the decrease in power output of a solar chimney power plant at high pressure drops may depend on a variety of factors, including the specific design and operating conditions of the system. It is important to carefully evaluate the effects of pressure drop and other factors on the performance of the SCPP to optimize the design and operation of the system for maximum power output and efficiency.

## Conclusion

In this article, a numerical investigation of a combined SCPP with gas power plant is performed. The outlet hot gas from gas power plant, instead of being released into the atmosphere from the stacks, flows through the buried pipes under the canopy and the energy of the outlet hot gas from gas power plant can be used to generate power. Different parameters such as the air temperature at the exhaust of chimney, lost heat through the canopy, lost heat at the outlet of chimney and output power from SCPP are studied in detail. It is found that increasing the turbine pressure ratio has two completely different effects on the energy wasted through the outlet of chimney and the canopy, so that increasing the turbine pressure drop reduces the energy wasted from the outlet of chimney and increases the heat losses through canopy. By burying the pipes in which hot gas flows, the wasted heat from the outlet of chimney and canopy increases. As the most important result of this study, it can be found that burying the pipes in which the outlet hot gas from the gas turbine flows leads to an increase in the output power of SCPP by 554%, 208% and 125% at the radiation flux of 200 W/m^2^, 500 W/m^2^ and 800 W/m^2^, respectively.

## Data Availability

All data generated or analysed during this study are included in this published article.
